# Evaluation of silicon strip detectors in transmission mode for online beam monitoring in microbeam radiation therapy at the Australian Synchrotron

**DOI:** 10.1107/S1600577521011140

**Published:** 2022-01-01

**Authors:** Jeremy Davis, Andrew Dipuglia, Matthew Cameron, Jason Paino, Ashley Cullen, Susanna Guatelli, Marco Petasecca, Anatoly Rosenfeld, Michael Lerch

**Affiliations:** a Centre for Medical Radiation Physics, University of Wollongong, Wollongong, New South Wales, Australia

**Keywords:** transmission detectors, beam monitoring, real-time dosimetry, microbeam radiation therapy

## Abstract

Successful transition of synchrotron-based microbeam radiation therapy (MRT) from pre-clinical animal studies to human trials is dependent upon ensuring that there are sufficient and adequate measures in place for quality assurance purposes. Transmission detectors provide researchers and clinicians with a real-time quality assurance and beam-monitoring instrument to ensure safe and accurate dose delivery. In this work, the effect of transmission detectors of different thicknesses (10 and 375 µm) upon the photon energy spectra and dose deposition of spatially fractionated synchrotron radiation is quantified experimentally and by means of a dedicated *Geant4* simulation study. The simulation and experimental results confirm that the presence of the 375 µm thick transmission detector results in an approximately 1–6% decrease in broad-beam and microbeam peak dose. The capability to account for the reduction in dose and change to the peak-to-valley dose ratio justifies the use of transmission detectors as thick as 375 µm in MRT provided that treatment planning systems are able to account for their presence. The simulation and experimental results confirm that the presence of the 10 µm thick transmission detector shows a negligible impact (<0.5%) on the photon energy spectra, dose delivery and microbeam structure for both broad-beam and microbeam cases. Whilst the use of 375 µm thick detectors would certainly be appropriate, based upon the idea of best practice the authors recommend that 10 µm thick transmission detectors of this sort be utilized as a real-time quality assurance and beam-monitoring tool during MRT.

## Abstract 2

This study provides an extensive evaluation of the suitability of transmission detectors for use in synchrotron-based microbeam radiation therapy applications.

## 1. Introduction

Synchrotron X-ray microbeam radiation therapy (MRT) is a promising radiotherapy modality for the treatment of neurological disorders and inoperable brain tumours, particularly those presenting in paediatric patients (Slatkin *et al.*, 1992 ▸; Dilmanian *et al.*, 2002 ▸; Laissue *et al.*, 2007 ▸; Bouchet *et al.*, 2010 ▸; Romanelli *et al.*, 2011 ▸). MRT makes use of highly collimated quasi-parallel X-ray microbeams, with typical widths ranging from 20 to 100 µm and pitch (*i.e.* centre-to-centre distance) ranging from 100 to 400 µm. This results in a dosimetric profile consisting of high dose rate ‘peaks’ which are separated by low dose rate ‘valleys’ formed primarily by scattered X-rays and secondary electrons generated within the ‘peak’ regions. The key advantage of MRT over traditional external beam radiotherapy techniques is the extraordinary radio-resistance demonstrated by normal tissue relative to cancerous tissue when irradiated by spatially fractionated micrometre-scale radiation fields (due to the dose–volume effect), allowing for a larger therapeutic dose delivery to the tumour site (Slatkin *et al.*, 1992 ▸; Laissue *et al.*, 1998 ▸, 1999 ▸, 2001 ▸; Regnard *et al.*, 2008 ▸; Crosbie *et al.*, 2010 ▸; Bouchet *et al.*, 2013 ▸; Bräuer-Krisch *et al.*, 2015 ▸; Engels *et al.*, 2020 ▸). In order to avoid complications, including dose smearing due to cardio-synchronous movement, which would negate gains in normal tissue radio-resistance (Duncan *et al.*, 2019 ▸), exceptionally high dose rates are required to be delivered with sub-millimetre precision in small time frames.

From a dosimetric perspective, the task of quality assurance (QA) for MRT is extremely complex and challenging. The key dosimetric parameters in MRT are related to the structure of the microbeam, such as the full width at half-maxiumum (FWHM) and the quality of the microbeam collimation, which can be evaluated by the peak-to-valley dose ratio (PVDR).

The combination of a high dose rate and spatial fractionation presents a unique challenge not present in conventional radiotherapy applications. The high dose rate of synchrotron-based MRT requires detectors with superior radiation hardness to enable long-term operation. The spatial fractionation and steep dose gradients that are unique to MRT require additional features of high spatial resolution and large dynamic range in order to measure both peak and valley doses accurately.

At present there are a limited number of detector systems which are able to fulfil the aforementioned requirements, with the most notable of these being solid state in nature, such as silicon-based MOSFETs, strip detectors (Rosenfeld *et al.*, 1999 ▸, 2005 ▸; Lerch *et al.*, 2011 ▸; Petasecca *et al.*, 2012 ▸; Fournier *et al.*, 2017 ▸; Davis *et al.*, 2018 ▸; Cameron *et al.*, 2019 ▸) and commercial diamond-based detectors (PTW, 2019 ▸; Livingstone *et al.*, 2016 ▸; Butler *et al.*, 2018 ▸; Davis *et al.*, 2019 ▸). Dosimetry protocols for synchrotron-based radiotherapy and synchrotron-based MRT can be found in the work presented by Prezado *et al.* (2011 ▸) and Davis *et al.* (2021 ▸), respectively.

Given the pre-clinical status of MRT, the technology, protocols and treatment planning systems associated with performing dosimetry for QA purposes are still in the developmental phase. Fortunately, this emergent modality is able to take advantage of well known and established methodologies utilized in clinical radiotherapy applications to guide the development and implementation of new technologies and practices for use in MRT. This study is illustrative of this idea, inasmuch as the purpose is related to the investigation of technologies to assume a comparable role to that of a CLINAC ion chamber in the measurement of ‘monitor units’ but to suit the vastly more complex paradigm of MRT. Whilst synchrotron-based therapeutic beamlines such as the Australian Synchrotron’s (AS) Imaging and Medical Beam-Line (IMBL) do utilize free air ionization chambers to monitor the beam intensity, this is deemed insufficient in terms of complying with the IAEA’s definition of QA in external beam radiotherapy. The IAEA states that QA must encompass all ‘necessary procedures that ensure consistency of the medical prescription, and safe fulfilment of that prescription, as regards the dose to the target volume, together with minimal dose to normal tissue, minimal exposure of personnel and adequate patient monitoring aimed at determining the end result of the treatment’ (IAEA, 1998 ▸). In mega-voltage (MV) applications on a CLINAC, this task of QA has inspired the development of new technologies and methods to extend the capability of medical physicists to monitor not only the intensity, but also the shape and size, which are of particular relevance in some of the more advanced techniques available in conventional radiotherapy, such as intensity-modulated radiation therapy (IMRT) and volumetric modulated arc therapy (VMAT), with notable examples by do Amaral *et al.* (2015 ▸) and by Matar *et al.* (2020 ▸). Both approaches utilize the LINAC accessory tray to support their chosen technology and each method is capable of performing personalized QA for patients, including the identification of possible sources of error during treatment. The 2D array of silicon diodes described by Matar *et al.* (2020 ▸) is representative of the parallel occurring between conventional radiotherapy and MRT, requiring small but important adjustments to be suitable to the context of MRT.

Like its conventional radiotherapy equivalent, outlined by Matar *et al.* (2020 ▸) for MV applications, a detector operating in transmission mode for MRT should incorporate an array structure with multiple-channel readout to allow for simultaneous measurement of both peak and valley doses. To be suitable for MRT, the detector should incorporate a 1D or 2D array of single-strip detectors with an appropriate pitch to account for the fractionated nature of the field, *i.e.* to allow for simultaneous measurement of peak and valley intensities. A multi-strip detector of this sort running in transmission mode will have the potential to detect deviations in the intensity and structure of the fractionated field during treatment delivery from that of the treatment plan to be detected and monitored and, if necessary, enact automatic beam shut-down procedures. In the case of the IMBL, the capability to connect a suitable detector directly to the control system of the fast safety shutter (FSS) enables personnel to limit exposure time to that of the FSS activation time, *i.e.* ∼15 to 20 ms (Livingstone *et al.*, 2017 ▸; Davis *et al.*, 2019 ▸). Such a setup would inherently link real-time beam monitoring to treatment QA and ensure the safety of an MRT patient as best practice dictates. The use of silicon-based transmission multi-strip detectors for beam monitoring at the European Synchrotron Radiation Facility (ESRF, Grenoble, France) has been explored extensively (Lerch *et al.*, 2017 ▸; Kalliopuska *et al.*, 2011 ▸; Bräuer-Krisch *et al.*, 2015 ▸; Povoli *et al.*, 2015 ▸). These studies have demonstrated the effectiveness of such detectors as online beam monitors for MRT. To date, two such multi-strip silicon-based transmission detectors have been developed and characterized (Kalliopuska *et al.*, 2011 ▸; Povoli *et al.*, 2015 ▸). Fundamentally, the designs of the two detectors are identical, with the only significant difference being the thickness (*i.e.* 375 or 10 µm for thick and thin monitors, respectively).

In this study, experimental measurements were performed at the AS in the presence and absence of the two afore­mentioned transmission detector versions, with each positioned upstream of a water phantom to quantify the impact their insertion has upon the dose deposition and structure of the fractionated treatment field. Experimental results were also compared against *Geant4* Monte Carlo simulations to provide a theoretical point of comparison and to assess the impact of the transmission detectors on the photon energy spectra.

## 2. Method

Experimental measurements were performed in Hutch 2B of the AS IMBL, which is positioned 32 m away from the 3 T superconducting multi-pole wiggler source. *In vacuo* filtration was used to moderate the photon flux and dose rate (Table 1 ▸). The reader is directed to the paper by Stevenson *et al.* (2016 ▸) for a detailed description of the AS IMBL and its components.

Dose measurements were performed within a 100 mm × 100 mm × 140 mm water phantom. Two different commercial detectors were used to provide dosimetric measurements in this work: the PTW PinPoint 31014 ionization chamber (IC) and the PTW microDiamond (PTW, 2019 ▸; Livingstone *et al.*, 2016 ▸; Davis *et al.*, 2019 ▸; Brace *et al.*, 2020 ▸). The PinPoint IC was used to cross-calibrate the PTW microDiamond under reference conditions, *i.e.* 20 mm depth and 20 mm × 20 mm field size, following a methodology presented by Davis *et al.* (2021 ▸). This methodology is based on an adaptation of the IAEA TRS-398 code of practice for a medium-energy kilovoltage X-ray beam for use with synchrotron-generated X-rays pertaining to use in MRT. A UNIDOS webline electrometer is used for readout of the integral dose after each translation of the detector through the field.

Percentage depth dose (PDD) plots in the broad-beam context are produced by normalizing the detector response to 100% (with or without the transmission detector in place) relative to the response of the detector at 20 mm depth in water without the transmission detector in place.

### 2.1. *Geant4* simulation study

The *Geant4-IMBL* simulation package, which has previously been validated for modelling X-ray fluence and energy spectra and subsequent dose distribution on the AS IMBL (Dipuglia *et al.*, 2019 ▸), was utilized in this study. The purpose of its use in this study was to determine how the presence of the transmission detector would affect the energy spectra, as well as the uncollimated broad-beam and collimated microbeam depth dose profiles.

**Figure 1:**
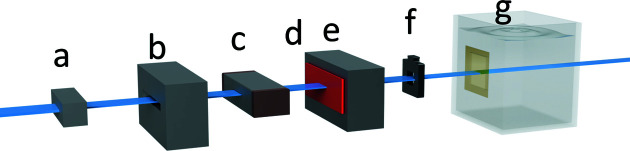
A schematic diagram representing the major components simulated. The beam (illustrated in blue) transits through, in order, **a** the beam-defining aperture, **b** the first open-air IC, **c** the multi-slit collimator, **d** the location where the silicon slab may be placed, **e** the second open-air IC, **f** the location of the confocal mask, and finally **g** the water phantom housing the detector.

In the simulation model, a silicon slab (red) representing the transmission detector may be defined immediately behind the multi-slit collimator (MSC) at position **d** in Fig. 1 ▸ within Hutch 2B of the IMBL. The MSC in the first half of this study is defined to be out of field to produce a broad-beam radiation field. Two different thicknesses (10 and 375 µm) of silicon were modelled to match the known specifications of the silicon transmission detectors used experimentally (Kalliopuska *et al.*, 2011 ▸; Povoli *et al.*, 2015 ▸). Phase space files (PSFs) were produced between positions **e** and **f** in Fig. 1 ▸ to contain information pertaining to the incident photon spectra (*e.g.* energy, momentum and polarization), positioned 0.9 m upstream from the surface of the water phantom (Cameron *et al.*, 2017 ▸). PSFs were produced with and without the transmission detectors defined, to allow for spectral comparison and determination of the effect of the detectors on the spectrum. In this study, as with the previous study by Dipuglia *et al.* (2019 ▸), the beam is considered to be travelling along the *x* axis, with the *y* and *z* axes considered as the horizontal and vertical axes, respectively.

The water phantom was modelled as a rectangular slab with dimensions of 140 mm × 100 mm × 100 mm (*x* × *y* × *z*) and voxelized in 2 mm × 2 mm × 5 mm elements for the broad-beam configuration. The voxel dimensions were chosen to match the dimensions of the PTW PinPoint IC in order to provide a basis for comparison. The voxelization was performed in order to measure energy deposition and provide a determination of dose as a function of depth in order to evaluate the effect of the transmission detector (TD) on the depth dose response. Lastly, the MSC was defined to be in-field to produce the microbeam configuration. The water phantom was again voxelized for the microbeam configuration, now in 1 mm × 0.01 mm × 0.1 mm elements to provide the required spatial resolution for a fractionated radiation field with 50 µm wide peaks and a centre-to-centre spacing of 400 µm. Using this level of spatial resolution allows for a comparison of peak and valley doses as a function of depth with and without the presence of the transmission detector in-field. Full details of the *G4IMBL* package can be found in the paper written by Dipuglia *et al.* (2019 ▸).

### 2.2. Broad-beam mode

**Figure 2:**
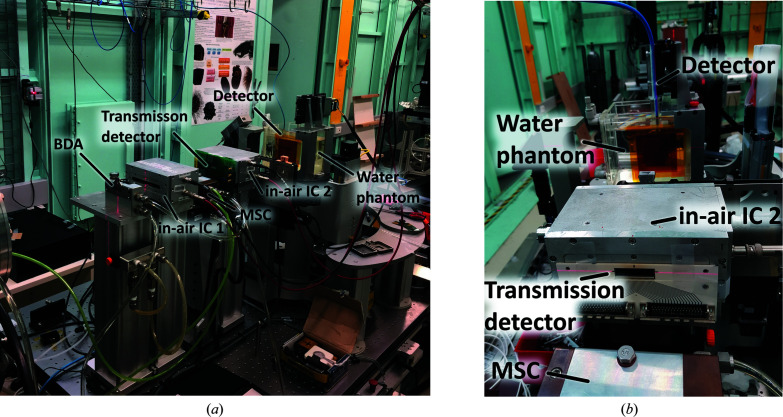
(Left) The experimental setup within Hutch 2B of the Imaging and Medical Beam Line at the Australian Synchrotron, featuring the 10 µm thick transmission detector in front of the second in-air ionization chamber, IC2. (Right) The experimental setup of the 375 µm thick transmission detector in front of the second in-air ionization chamber.

Two different broad-beam fields were utilized in this part of the study depending on whether the 375 or 10 µm thick transmission detector was currently set up within field. Different field sizes were required to limit photon interaction with the silicon-based detectors and avoid photon interactions with the high-*Z* components of the attached PCB. In the case of the 375 µm thick transmission detector, in order to shape and confine the field to the required size, high-precision (∼10 µm) ‘shaping’ and ‘clean-up’ slits positioned upstream of the sample stage, in combination with a 30 mm × 1 mm beam-defining aperture (BDA) inside the experimental hutch, were used to define an intrinsic broad-beam field size of 25 mm × 1 mm. The transmission detector previously described was placed upstream of the phantom (Fig. 2 ▸), after the MSC and immediately before the second in-air ionization chamber. A tungsten confocal mask (position **f** in Fig. 1 ▸) with a 20 mm × 20 mm aperture was used to limit roll off and further define the intrinsic field size incident upon the phantom. Finally, a 20 mm × 20 mm ‘treatment field’ was produced by vertically translating the phantom and mask through the intrinsic field.

In the case of the 10 µm thick transmission detector, the field size was further reduced to limit the field to be solely within the window of the silicon detector and not to interact with the high-*Z* components of the attached PCB. Using the slits, the horizontal field size was reduced from 25 to 15 mm. The vertical field size was further reduced to 0.5 mm using the 30 mm × 0.5 mm BDA. The field size was further defined with the use of a tungsten conformal mask with a 10 mm × 10 mm aperture. Similar to the previous setup, a 10 mm × 10 mm ‘treatment field’ was produced by vertically translating the phantom through the intrinsic field.

Once calibrated, the PTW PinPoint IC was aligned to the centre of the intrinsic field in terms of the relative horizontal and vertical motor positions, as determined by maximizing the detector signal amplitude. Depth dose measurements were performed both with and without the 375 µm thick and 10 µm thick transmission detectors in place by vertically scanning the PTW PinPoint IC through the intrinsic field at depths ranging from 5 to 75 mm within the water phantom.

### 2.3. Microbeam mode

Following insertion of the MSC, the intrinsic microbeams were measured with the PTW microDiamond and X-Tream dosimetry data acquisition system (Petasecca *et al.*, 2012 ▸; Davis *et al.*, 2019 ▸; Cameron *et al.*, 2019 ▸). The X-Tream data acquisition system has a sampling rate of 1 MHz, allowing for data acquisition of the dose in 1 µm intervals. Intrinsic microbeam profiles were acquired by scanning the vertically aligned detector horizontally through the field at a depth of 20 mm within the water phantom. Intrinsic microbeam profiles were used to measure the position of the central microbeams relative to the DynMRT stage.

Following this determination of the spatial positioning, the central microbeam and adjacent valleys were then measured in step-and-scan (SnS) mode with the PTW microDiamond using the PTW UNIDOS webline electrometer. SnS mode in this instance refers to stepping horizontally with respect to the incident beam prior to scanning vertically through the field at a constant scan speed. Given appropriate knowledge of the microbeam position, the step size was adjusted to 20 µm within the valleys and 5 µm in the peaks. The valley dose was determined by averaging the dose over the central 100 µm of a valley adjacent to the central microbeam peak. This averaging technique is utilized in order to minimize the effects of any internal structure present within the valleys, such as that caused by small-angle scattering effects. SnS profiles were measured at 5, 10 and 75 mm depths in order to evaluate the PVDR as a function of depth. This measurement also provides valuable information related to the changing peak dose and valley dose characteristics as a function of depth.

## 3. Results and discussion

### 3.1. *Geant4* simulation study

**Figure 3:**
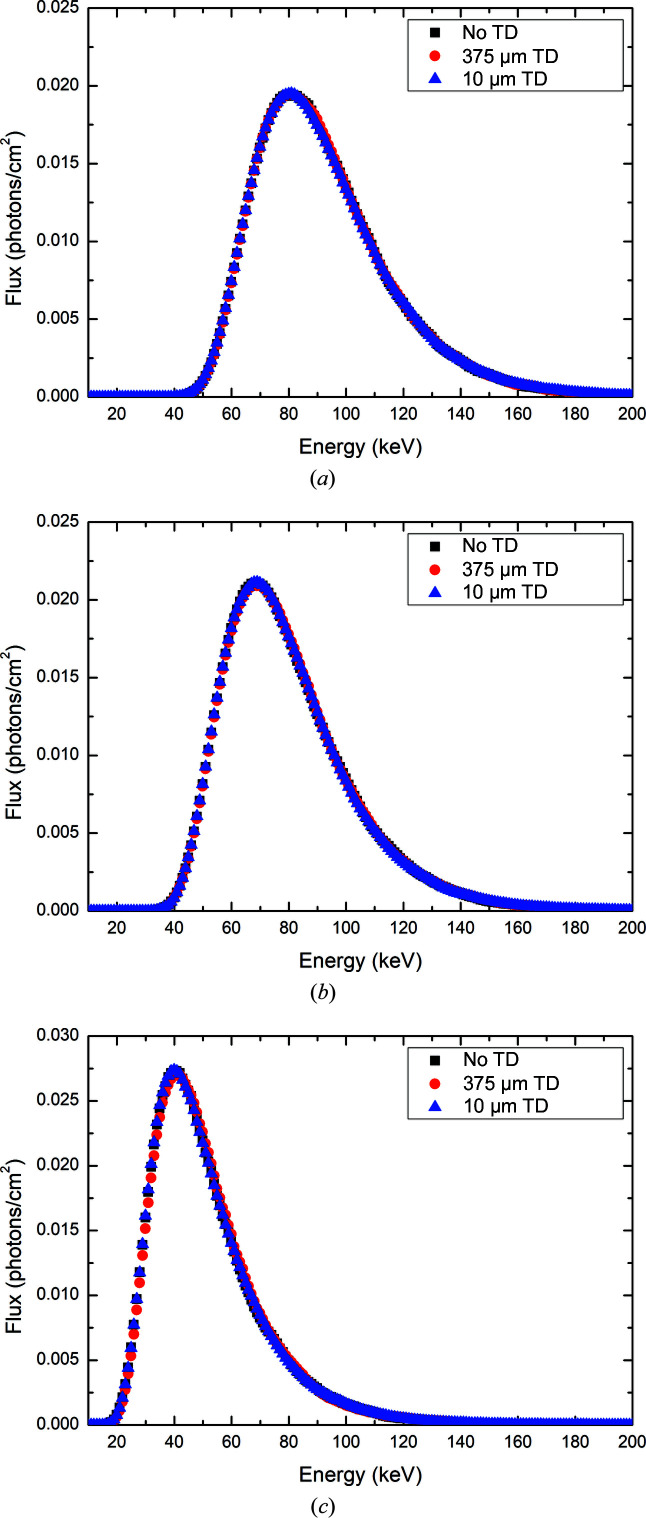
Simulated energy spectra in 3 T mode with (*a*) Cu/Cu filtration, (*b*) Cu/Al filtration and (*c*) Al/Al filtration. The spectra in the absence of the transmission detectors (TDs) (black) are compared with the spectra downstream of the thick (red) and thin (blue) transmission detectors.

The results presented in this section were derived from the *Geant4-IMBL* simulation package described by Dipuglia *et al.* (2019 ▸). This model has been extensively characterized and validated with respect to the analytic model developed by Stevenson *et al.* (2016 ▸) and shows excellent agreement with the commonly used software *SPECTRA* (Tanaka & Kitamura, 2001 ▸) and *XOP* (Sanchez del Rio & Dejus, 2011 ▸) and experimental results. The synchrotron X-ray spectra simulated by the *G4IMBL* simulation were stored in a PSF situated before the water phantom in hutch 2B, after transport through the IMBL. The PSF records all pertinent information related to simulated photons, including position, momentum, polarization, fluence and energy. The energy spectra (binned with an energy resolution of 0.1 keV) of the synchrotron X-rays for three different filtration combinations are presented in Fig. 3 ▸. A comparison of the PSF, *i.e.* X-ray energy spectra, with and without the 375 µm thick or 10 µm thick transmission detectors in place, allows for a determination of how the photon field has changed in terms of the mean and maximum energies (Table 2 ▸).

It is clear from the results presented in Table 2 ▸ and Fig. 3 ▸ that the 10 µm thick transmission detector has a negligible effect (≤0.3%) upon the mean and maximum energies of the transmitted photons through to the phantom downstream for each of the three photon fields investigated. In contrast, the presence of the 375 µm thick transmission detector in-field causes a measurable perturbation of the lower-energy photons in the X-ray spectra, thus hardening the beam, reflected by the increase in the maximum and mean energies. The most significant effect observed is an approximate 5% change in the maximum energy for the 3 T Al/Al filtration mode, compared with the approximately 1.2% and 1.5% changes observed for the 3 T Cu/Al and Cu/Cu filtration modes, respectively.

**Figure 4:**
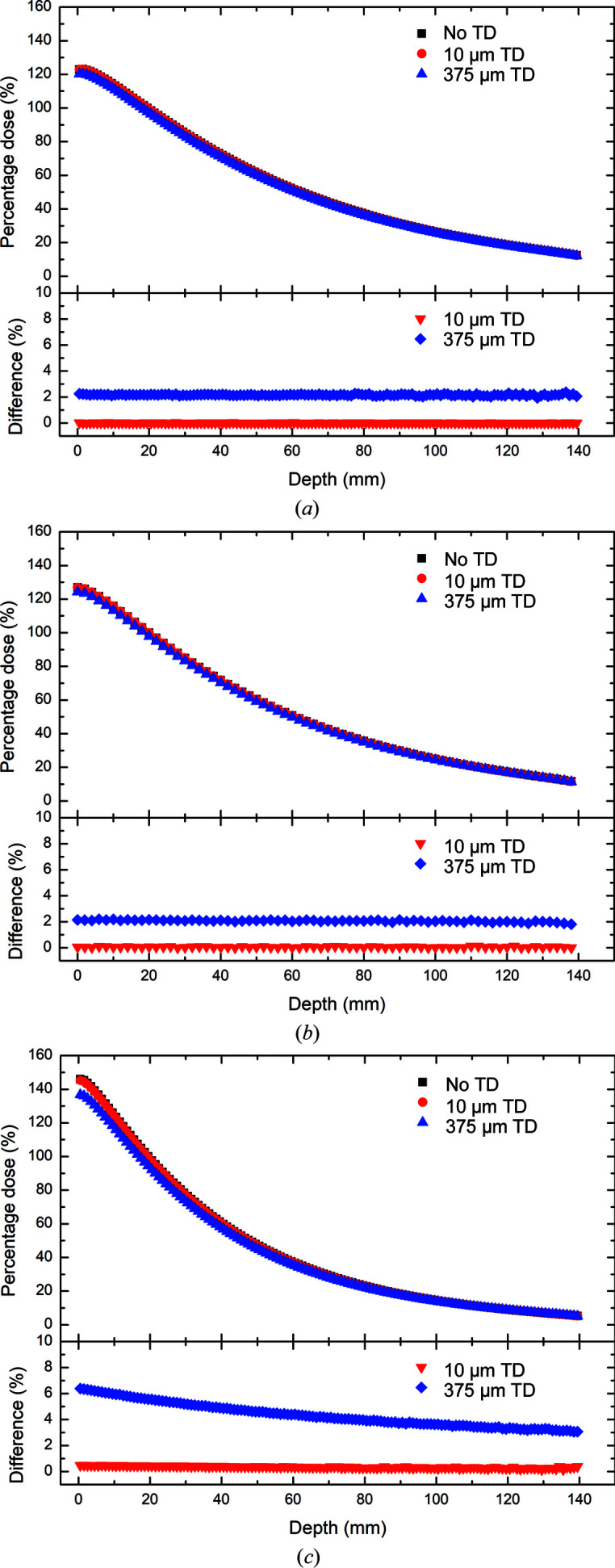
Simulated percentage depth doses in 3 T mode with (*a*) Cu/Cu filtration, (*b*) Cu/Al filtration and (*c*) Al/Al filtration. The percentage depth doses in the absence of the transmission detectors (TDs) (black) are compared with the percentage depth doses downstream of the 10 µm thick (red) and 375 µm thick (blue) transmission detectors. The percentage differences between the response with and without the 10 µm thick (red) and 375 µm thick (blue) transmission detectors in front of the water phantom are presented below each percentage depth dose plot.

The percentage depth doses and percentage differences as a function of depth with and without the 375 or 10 µm thick transmission detectors for Cu/Cu, Cu/Al and Al/Al are shown in Fig. 4 ▸. The percentage depth dose curves with and without the transmission detectors in place portray the expected exponential decay typical for attenuated photons in a uniform medium. The presence of the 375 µm thick transmission detector results in a small but noticeable attenuation of the photon field, leading to a discernible decrease in dose at each depth. As expected, the decrease in dose due to attenuation within the transmission detector shows a dependence upon the beam quality or energy. Average decreases in dose of (2.16 ± 0.06)%, (2.06 ± 0.07)% and (4.34 ± 0.93)% due to the presence of the 375 µm thick transmission detector are observed for the 3 T Cu/Cu, Cu/Al and Al/Al photon fields, respectively. In contrast, the 10 µm-thick transmission detector has a negligible effect (<1%) on the average delivered dose for all three modalities across all depths, with average decreases in dose of (0.01 ± 0.01)%, (0.05 ± 0.03)% and (0.35 ± 0.07)% for the 3 T Cu/Cu, Cu/Al and Al/Al fields, respectively.

**Figure 5:**
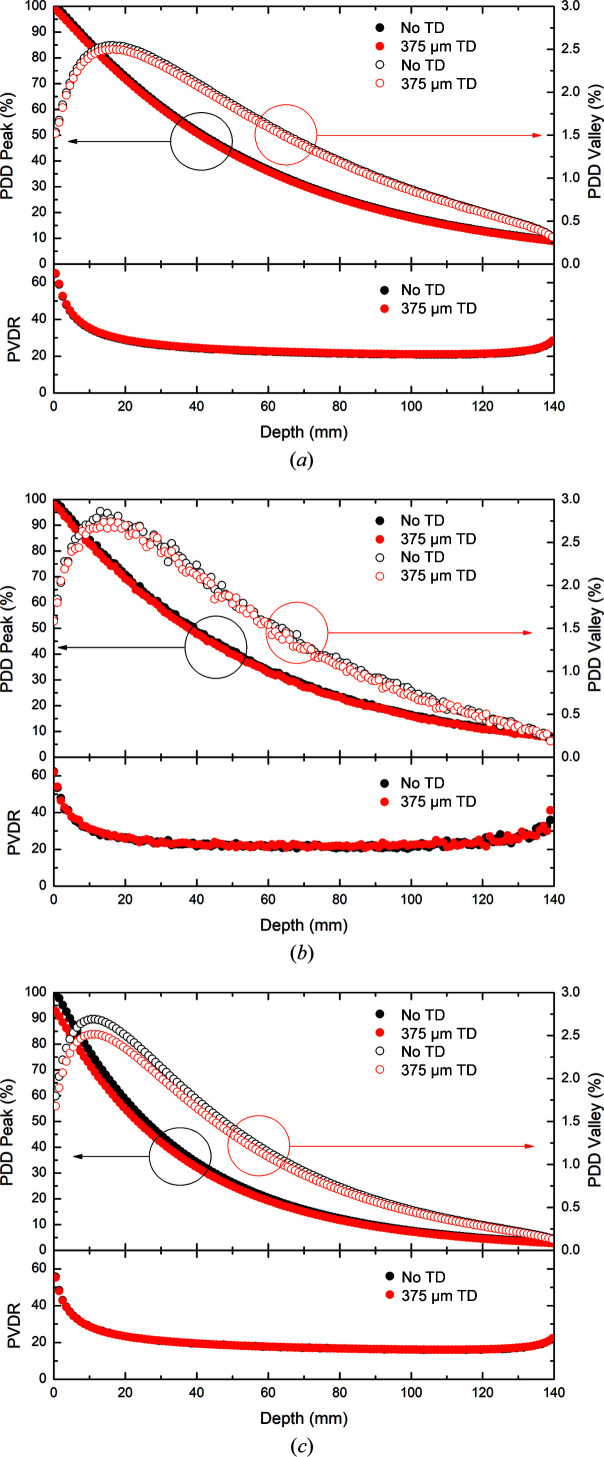
Simulated peak (solid symbols) and valley (open symbols) doses and PVDR (bottom plots) as a function of depth in a water phantom in 3 T mode with (*a*) Cu/Cu filtration, (*b*) Cu/Al filtration and (*c*) Al/Al filtration.

Fig. 5 ▸ depicts the peak and valley doses as a function of depth for the 3 T Cu/Cu, Cu/Al and Al/Al photon fields within a water phantom, with and without the transmission detectors in-field. Like the broad-beam case in Fig. 4 ▸, the peak dose within the phantom follows the typical depth dose response as the photon beam is attenuated. The average decreases in the peak dose with depth due to the 375 µm thick transmission detector are (1.34 ± 0.05)%, (2.03 ± 1.31)% and (5.19 ± 1.06)%, respectively, for the 3 T Cu/Cu, Cu/Al and Al/Al photon fields.

The valley dose response curve as a function of depth differs from that of the peak dose due to the underlying mechanisms responsible. The valley dose is caused primarily through secondary electron production within the peak regions, with these electrons traversing into the adjacent valleys, and also through the scattering of photons within the water phantom medium and, to a lesser extent, through small-angle scattering in the MSC. The mechanism behind the origin of the valley dose inherently provides an explanation of the build-up region in the valley dose response curve, as well as of the initial decrease in the PVDR. After the peak in the valley dose occurs, we see a decaying response curve similar to that of the peak but with some notable differences, such as the shallow gradient of the decay curve and the rapid fall off within the last ∼10 mm. These features can be explained by the increasing lack of medium as the end of the phantom is approached, which limits back scattering and secondary electron production. These regions also correspond to the relative plateau and eventual increase observed in the PVDR.

The average decreases in the valley dose with depth due to the insertion of the 375 µm thick transmission detector are (1.61 ± 0.28)%, (2.94 ± 5.09)% and (5.23 ± 0.95)%, respectively, for the 3 T Cu/Cu, Cu/Al and Al/Al photon fields. Fig. 5 ▸ also depicts the PVDR as a function of depth for the 3 T Cu/Cu, Cu/Al and Al/Al photon fields within the water phantom, with and without the transmission detectors in-field. The results show increases in the average PVDR of (0.35 ± 0.08)%, (0.91 ± 5.25)% and (0.04 ± 0.15)%, respectively, for the 3 T Cu/Cu, Cu/Al and Al/Al photon fields are observed with the 375 µm transmission detector in place.

### 3.2. Broad-beam mode

**Figure 6:**
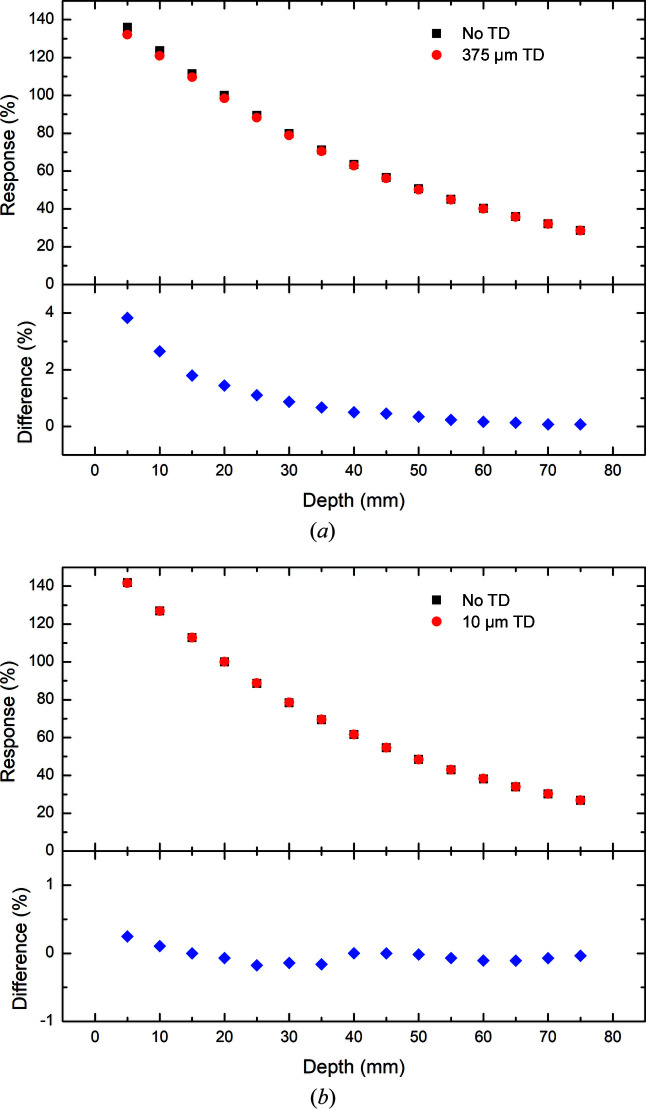
Depth dose measurements using the PTW PinPoint IC in the water phantom in 3 T mode with Al/Al filtration. The measurements are performed with (red) and without (black) (*a*) the 375 µm thick and (*b*) the 10 µm thick transmission detector (TD) in place. The percentage differences between the results with and without a transmission detector are presented below each plot.

Percentage depth dose (PDD) plots with and without the 375 µm thick and 10 µm thick transmission detectors in place measured with the PTW PinPoint IC for 3 T Al/Al, Cu/Al and Cu/Cu are presented in Figs. 6 ▸, 7 ▸ and 8 ▸. It should be reiterated here that the field definition (width and height) used in the investigation of the 375 µm thick transmission detector differs from that used to investigate the 10 µm thick transmission detector. In both cases, the results are normalized to 100% at 20 mm depth in water without the transmission detector in place. A summary of the results is presented in Tables 3 ▸ and 4 ▸ detailing the minimum, maximum and average percentage differences in response measured by the PTW PinPoint IC inside the phantom with and without either the 375 or 10 µm transmission detector inserted upstream of the water phantom.

In each filtration mode explored (3 T Al/Al, Cu/Al and Cu/Cu), the same decrease in response (percentage dose) as a function of depth is observed with and without the 375 µm thick transmission detector in place as with the simulation results. In comparison, the percentage depth dose plots depicted in Figs. 6 ▸, 7 ▸ and 8 ▸ show some notable differences from their simulation counterparts. Firstly, each of the experimental results shows the same decrease in deviation between the results with and without the 375 µm thick transmission detector. This deviation was previously observed with the simulation results in the 3 T Al/Al filtration mode, but was not apparent in the 3 T Cu/Al or Cu/Cu filtration modes, probably due to the increased statistical uncertainty at larger depths.

The results associated with the investigation of the 10 µm thick transmission detector show obvious differences from those obtained with the 375 µm thick transmission detector in terms of the percentage difference in response at different depths. In each of the three filtration modes investigated in this work, the magnitude of the percentage difference in response is relatively small, with the largest percentage difference between the responses with and without the 10 µm thick transmission detector observed at 15 mm depth with the 3 T Cu/Al filtration mode (Table 3 ▸). The average percentage differences as a function of depth for both the 10 and 375 µm thick transmission detectors are reported in Table 4 ▸. Regardless of the filtration mode, the percentage difference fluctuates slightly (± 0.2%) about the average which is approximately equal to zero. The results for the response measured by the PTW PinPoint IC with and without the 10 µm thick transmission detector in place are less than 0.25% across all depths of interest in this investigation, indicating a negligible impact upon the photon fluence and energy spectra due to the presence of the 10 µm thick transmission detector.

The percentage differences between measurements with and without the 375 µm thick transmission detector at 20 mm depth are represented in Table 5 ▸. For comparison, the experimental results derived from measurements with the PTW PinPoint IC are compared against the simulation results. Apart from the results obtained by the PTW PinPoint IC in the 3 T Al/Al filtration mode, which are probably due to the dose-rate dependence of the detector, the percentage differences predicted by the simulation are within 1% of those of the experiment. This good agreement between the experimental and simulation results is indicative of the validity of the methodology employed in this study.

### 3.3. Microbeam mode

Measured profiles of synchrotron-generated microbeams are presented in this section. The microbeam profiles were acquired with the PTW microDiamond in edge-on mode using the methodology outlined by Davis *et al.* (2019 ▸). SnS measurements of the central microbeam was performed at 5, 10 and 75 mm depth with and without the 375 µm thick transmission detector upstream.

**Figure 9:**
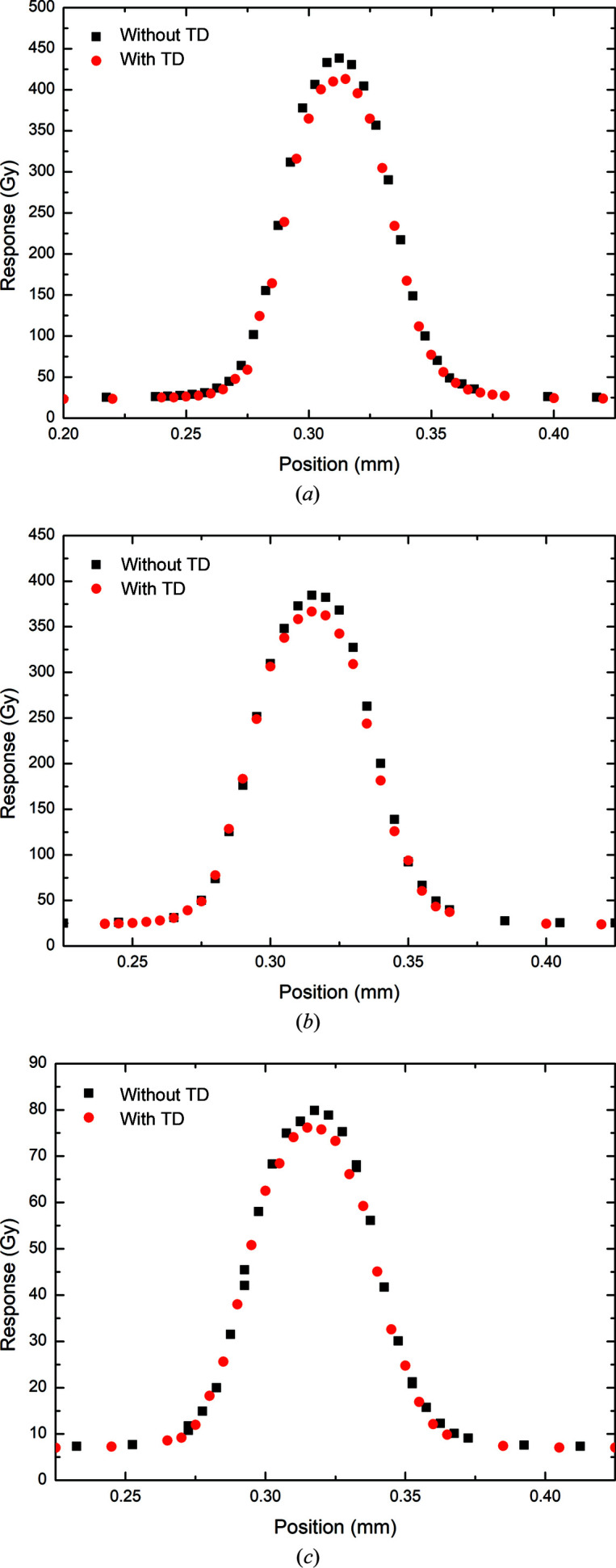
SnS profiles of the central microbeams measured with and without a transmission detector (TD) by the PTW microDiamond for 3 T Al/Al at (*a*) 5 mm depth, (*b*) 10 mm depth and (*c*) 75 mm depth.

In the 3 T Al/Al configuration at 5 mm depth, the percentage decreases in peak and valley doses due to the 375 µm thick transmission detector as measured by the PTW microDiamond were determined to be 5.83 and 6.32%, respectively (Fig. 9 ▸ and Table 6 ▸). As with the simulation results, the PVDR was shown to be higher with the transmission detector in place (18.34) than without (18.25), representing a difference of −0.53%. The trend continues at 10 mm depth, with 4.63 and 6.21% decreases in the peak and valley doses, respectively (Fig. 9 ▸). Likewise, at 75 mm depth decreases of 4.65 and 1.03% in the peak and valley doses, respectively, are measured (Fig. 9 ▸). The difference in the PVDR at increasing depth within the water phantom becomes much smaller, with differences of −1.69 and 3.66%, respectively, at depths of 10 and 75 mm.

**Figure 10:**
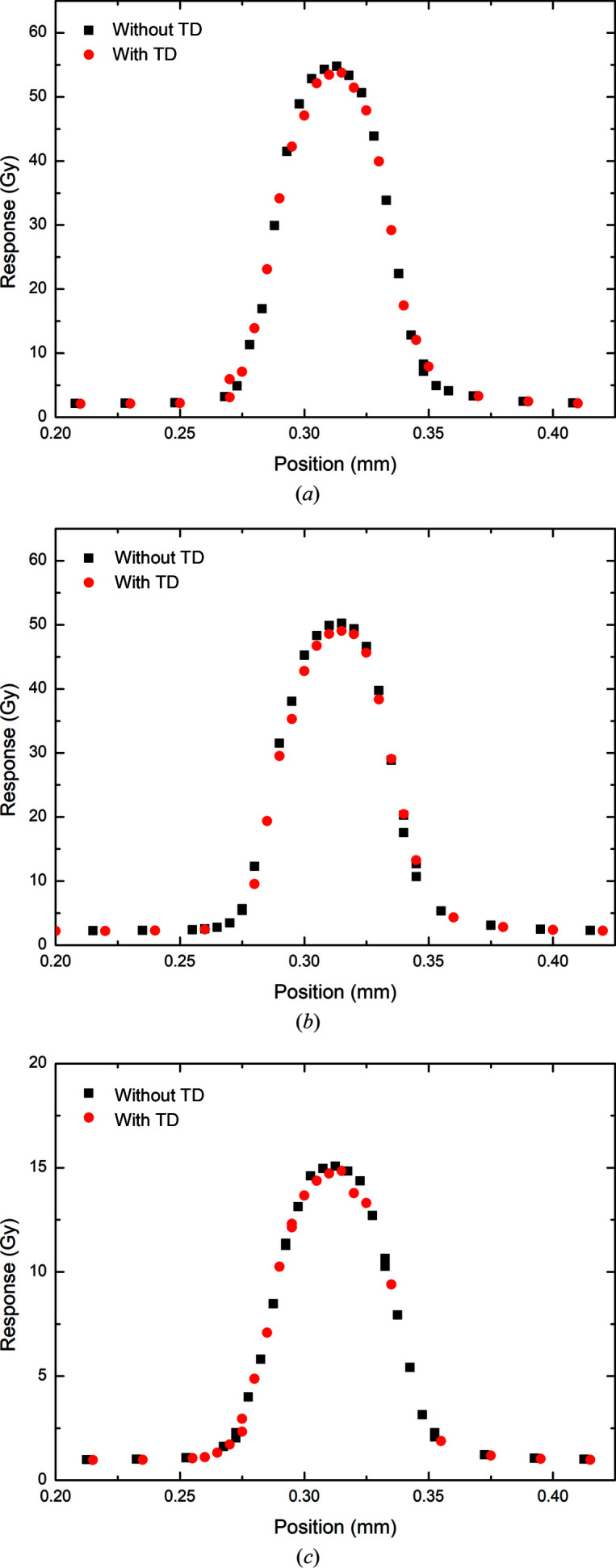
SnS profiles of the central microbeams measured with and without a transmission detector (TD) by the PTW microDiamond for 3 T Cu/Al at (*a*) 5 mm depth, (*b*) 10 mm depth and (*c*) 75 mm depth.

In the 3 T Cu/Al configuration at 5 mm depth, the percentage decreases in peak and valley doses due to the 375 µm thick transmission detector as measured by the PTW microDiamond were determined to be 1.82 and 5.70%, respectively (Fig. 10 ▸ and Table 7 ▸). As with the simulation results, the PVDR was shown to be higher when the transmission detector was inserted (26.53) than without (25.48), representing a decrease of −4.12%. The trend continues at 10 mm depth, with 2.34 and 3.08% decreases in the peak and valley doses, respectively (Fig. 10 ▸). Likewise, at 75 mm decreases of 1.56 and 2.26% in the peak and valley doses, respectively, are measured (Fig. 10 ▸). The difference in the PVDR at increasing depth within the water phantom becomes much smaller, with differences of −0.76 and −0.72%, respectively, at 10 and 75 mm.

**Figure 11:**
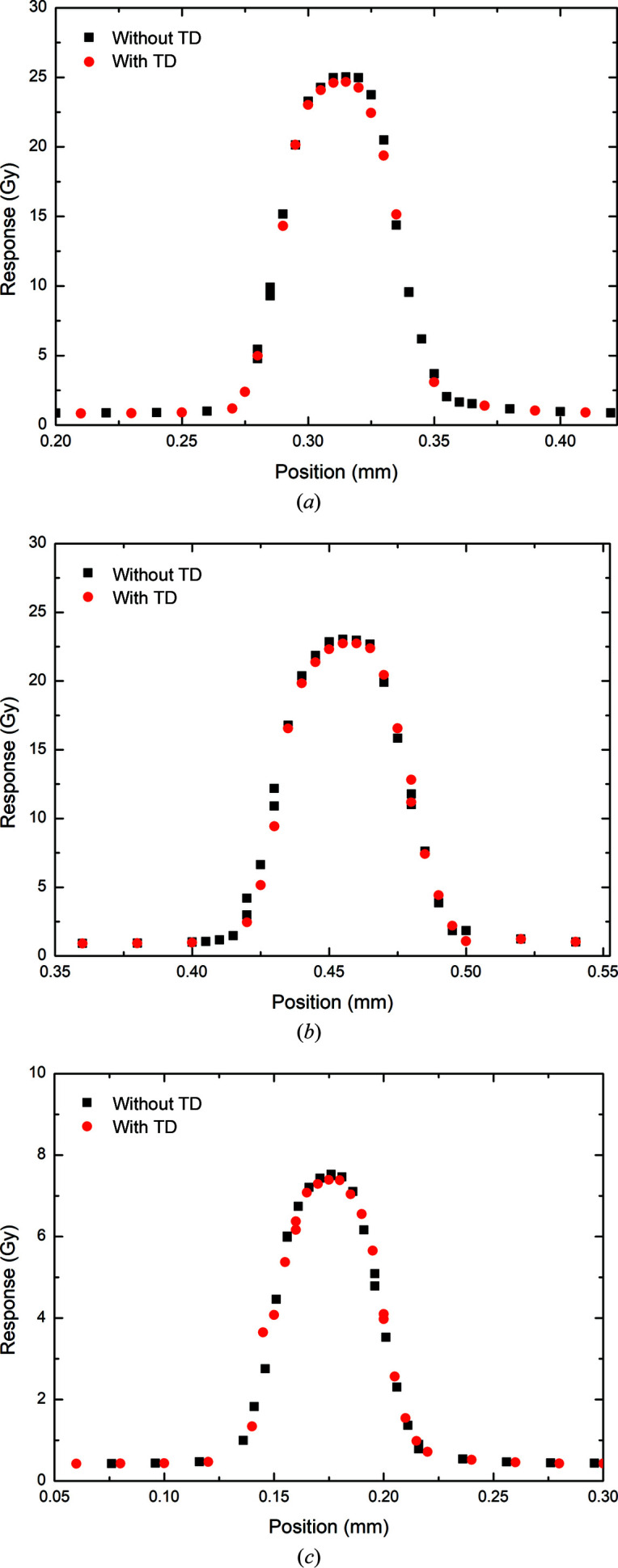
SnS profiles of the central microbeams measured with and without a transmission detector (TD) by the PTW microDiamond for 3 T Cu/Al at (*a*) 5 mm depth, (*b*) 10 mm depth and (*c*) 75 mm depth.

In the 3 T Cu/Cu configuration at 5 mm depth, the percentage decreases in peak and valley doses due to the 375 µm thick transmission detector as measured by the PTW microDiamond were determined to be 1.41 and 2.07%, respectively (Fig. 11 ▸ and Table 8 ▸). As with the simulation results, the PVDR was shown to be higher with the transmission detector in place (29.57) than without (29.37), a −0.68% difference. The trend continues at 10 mm depth, with 0.52 and 1.24% decreases in the peak and valley doses, respectively (Fig. 11 ▸). Likewise, at 75 mm decreases of 1.72 and 1.40% in the peak and valley doses, respectively, are measured (Fig. 11 ▸). The difference in the PVDR at increasing depth within the water phantom becomes much smaller, with differences of −0.73 and 0.32%, respectively, at 10 and 75 mm.

Determination of the FWHM value of the microbeam in SnS mode is achieved by fitting a Gaussian fitting function to the data points within the peak in Figs. 9 ▸, 10 ▸ and 11 ▸. The FHWMs, with or without the transmission detector in place, for 5, 10 and 75 mm depths are reported in Tables 6 ▸–8 ▸ ▸. Whilst the values are less than the expected value of 50 µm, probably due to the alignment of the PTW microDiamond, the deviation is deemed insignificant. The magnitude of the PVDR with depth, whilst different from the simulated results, does however follow the same expected trend.

## 4. Conclusions

Monte Carlo simulations and experimental measurements were performed to determine the effect of transmission detectors upon quality assurance (QA) for microbeam radiation therapy (MRT). Broad-beam and microbeam experimental measurements were performed using the PTW PinPoint IC and microDiamond within a water phantom in hutch 2B of the AS IMBL. A dedicated *Geant4* simulation was used to replicate the experimental conditions and provide complementary results for comparison.

The results derived from both the simulation and experimental studies confirm that the presence of the 375 µm transmission detector results in an approximately 1–6% decrease in broad-beam and microbeam peak dose, depending upon the filtration configuration utilized. A similar, if slightly less significant, decrease is observed within the valley dose, such that there is an overall increase in the PVDR at shallower depths. Whilst there are some changes to the magnitude of the dose and to the PVDR, there does not appear to be any noticeable change to the microbeam structure, *i.e.* no significant change in FWHM (≤ 3.85%).

The capability to account for the effect upon the PVDR in addition to the negligible effect upon the FWHM justify the use of transmission detectors as thick as 375 µm in MRT, provided that treatment planning systems are able to account for their presence and provide suitably corrected results. In addition, the increase in the PVDR might also be considered to be an argument in favour of the use of transmission detectors as a QA instrument. If, instead, the criterion for application of the transmission detector is defined to be a minimal perturbation of the field, then the discussion should be focused on transmission detectors that are as thin as reasonably possible, but not so thin that the magnitude of response due to radiation and that of electronic noise are comparable.

The results of this work derived from a combination of simulation and experimental investigations of a 10 µm thick transmission detector indicate no significant effect upon the QA parameters that could be measured in a clinically relevant manner. The authors recommend that transmission detectors of this sort be utilized as a real-time QA and beam-monitoring tool during MRT treatment.

## Figures and Tables

**Figure 7:**
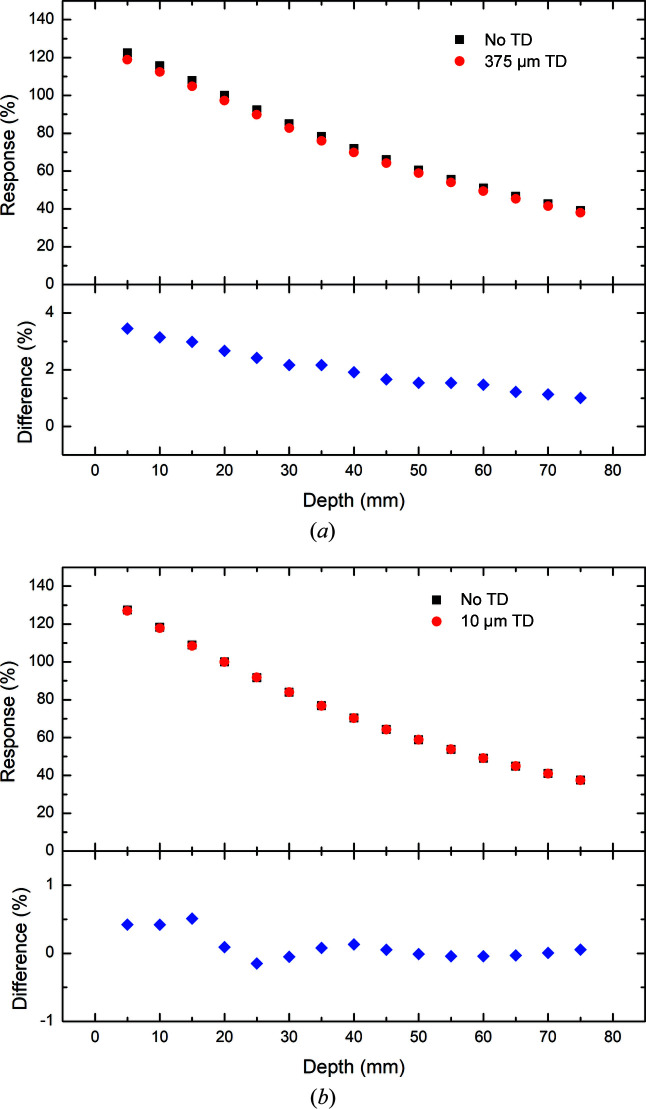
Depth dose measurements using the PTW PinPoint IC in the water phantom in 3 T mode with Cu/Al filtration. The measurements are performed with (red) and without (black) (*a*) the 375 µm thick and (*b*) the 10 µm thick transmission detector (TD) in place. The percentage differences between the results with and without a transmission detector are presented below each plot.

**Figure 8:**
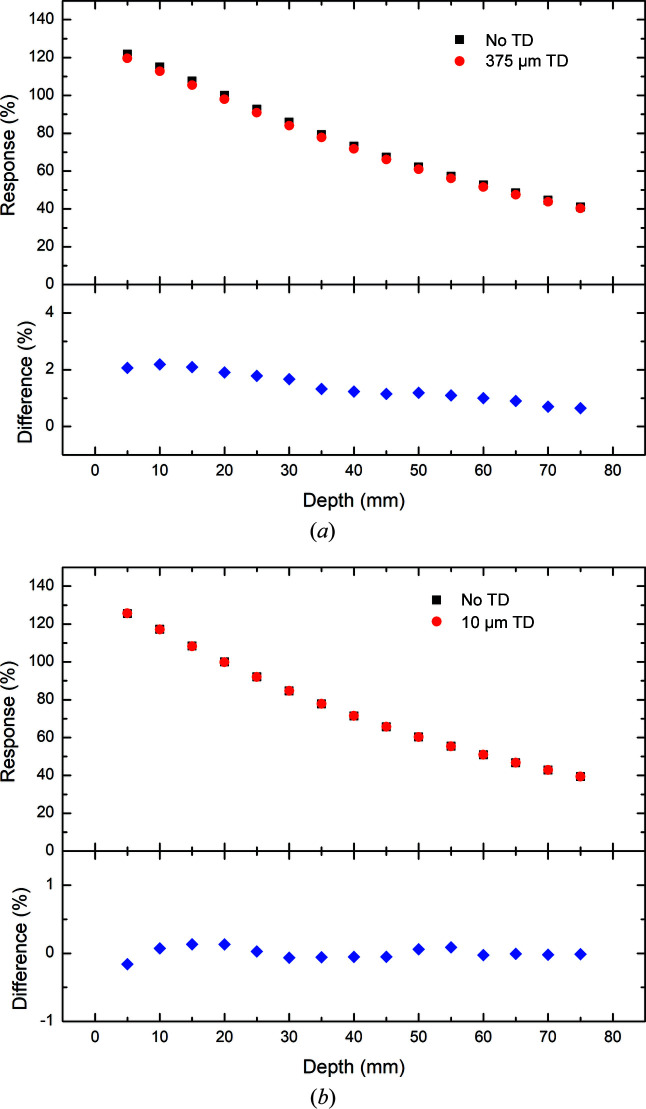
Depth dose measurements using the PTW PinPoint IC in the water phantom in 3 T mode with Cu/Cu filtration. The measurements are performed with (red) and without (black) (*a*) the 375 µm thick and (*b*) the 10 µm thick transmission detector (TD) in place. The percentage differences between the results with and without a transmission detector are presented below each plot.

**Table 1:** Relevant experimental parameters for the setup on the IMBL

Parameter	Filtration mode 1 (Cu/Cu)	Filtration mode 2 (Cu/Al)	Filtration mode 3 (Al/Al)
Electron energy in storage ring (GeV)	3.032	3.032	3.032
Storage ring current (mA)	200.2	200.2	200.2
Wiggler magnetic field strength (peak, T)	3	3	3
*In-vacuo* filtration	14.14 mm C (hd)	14.14 mm C (hd)	14.14 mm C (hd)
	+ 0.64 mm C	+ 0.64 mm C	+ 0.64 mm C
	+ 0.35 mm Be	+ 0.35 mm Be	+ 0.35 mm Be
	+ 1.41 mm Cu	+ 1.41 mm Cu	+2.83 mm Al
	+ 1.41 mm Cu	+ 2.83 mm Al	+2.83 mm Al
Dose rate (Gy s^−1^)	∼350	∼530	∼3250

**Table 2:** Relevant criteria for the synchrotron radiation spectra in 3 T with different filtration modes An energy threshold of 20–500 keV is applied to the data set with a bin size of 0.1 keV. TD stands for transmission detector.

Criterion	Mode	Without TD	With 10 µm TD	With 375 µm TD
Maximum energy (keV)	Al/Al	39.9 ± 0.1	39.9 ± 0.1	41.9 ± 0.1
Mean energy (keV)	Al/Al	35.2 ± 0.1	35.1 ± 0.1	35.4 ± 0.1
Maximum energy (keV)	Cu/Al	67.9 ± 0.1	67.9 ± 0.1	68.9 ± 0.1
Mean energy (keV)	Cu/Al	61.9 ± 0.1	61.9 ± 0.1	62.0 ± 0.1
Maximum energy (keV)	Cu/Cu	80.9 ± 0.1	80.9 ± 0.1	79.9 ± 0.1
Mean energy (keV)	Cu/Cu	71.1 ± 0.1	71.2 ± 0.1	71.3 ± 0.1

**Table 3:** Summary of experimental results for a broad-beam field for 3 T Al/Al, Cu/Al and Al/Al filtration modes The results show the percentage difference in response with and without the 375 or 10 µm transmission detector (TD) inserted upstream of the water phantom.

	375 µm TD		10 µm TD	
	Min % difference	Max % difference	Min % difference	Max % difference
Al/Al	0.08 (75 mm depth)	3.83 (5 mm depth)	−0.18 (25 mm depth)	0.25 (5 mm depth)
Cu/Al	1.00 (75 mm depth)	3.45 (5 mm depth)	−0.15 (25 mm depth)	0.53 (15 mm depth)
Cu/Cu	0.65 (75 mm depth)	2.60 (5 mm depth)	−0.16 (5 mm depth)	0.14 (15 mm depth)

**Table 4:** Summary of experimental results for a broad-beam field for 3 T Al/Al, Cu/Al and Al/Al filtration modes The results show the percentage difference in response with and without the 375 or 10 µm transmission detector (TD) inserted upstream of the water phantom.

	375 µm TD	10 µm TD
	Average % difference	Average % difference
Al/Al	0.96 ± 1.08	−0.04 ± 0.11
Cu/Al	2.03 ± 0.77	0.10 ± 0.20
Cu/Cu	1.40 ± 0.52	0.00 ± 0.08

**Table 5:** Difference in measured and simulated dose at 20 mm depth with and without the 375 µm thick transmission detector

Mode	Experimental (IC)	Simulation
Al/Al	1.44%	5.55%
Cu/Al	2.67%	2.15%
Cu/Cu	1.91%	2.2%

**Table 6:** FWHM, peak/valley dose and PVDR for microbeams at 5, 10 and 75 mm depth with and without the 375 µm transmission detector (TD) in place upstream Measurements are performed in the water phantom using the PTW microDiamond in 3 T mode with Al/Al filtration.

	5 mm depth			10 mm depth			75 mm depth		
Parameter	With TD	Without TD	Difference (%)	With TD	Without TD	Difference (%)	With TD	Without TD	Difference (%)
FWHM (µm)	45.5	46.8	2.78%	46.1	45.8	−0.66%	48.4	47.9	−1.04%
Peak dose (Gray)	412.89	438.45	5.83	366.71	384.50	4.63	76.16	79.87	4.65
Valley dose (Gray)	22.51	24.03	6.32	22.59	24.09	6.21	6.94	7.01	1.03
PVDR	18.34	18.25	−0.53	16.23	15.96	−1.69	10.98	11.39	3.66

**Table 7:** FWHM, peak/valley dose and PVDR for microbeams at 5, 10 and 75 mm depth with and without the 375 µm transmission detector (TD) in place upstream Measurements are performed in the water phantom using the PTW microDiamond in 3 T mode with Al/Al filtration.

	5 mm depth			10 mm depth			75 mm depth		
Parameter	With TD	Without TD	Difference (%)	With TD	Without TD	Difference (%)	With TD	Without TD	Difference (%)
FWHM (µm)	45.4	44.6	−1.79%	46.4	44.9	−3.34%	48.7	48.2	−1.04%
Peak dose (Gray)	53.78	54.77	1.82%	49.06	50.24	2.34%	14.84	15.08	1.56%
Valley dose (Gray)	2.03	2.15	5.70%	2.18	2.25	3.08%	0.94	0.96	2.26%
PVDR	26.53	25.48	−4.12%	22.47	22.29	−0.76%	15.77	15.66	−0.72%

**Table 8:** FWHM, peak/valley dose and PVDR for microbeams at 5, 10 and 75 mm depth with and without the 375 µm transmission detector (TD) in place upstream Measurements are performed in the water phantom using the PTW microDiamond in 3 T mode with Cu/Cu filtration.

	5 mm depth			10 mm depth			75 mm depth		
Parameter	With TD	Without TD	Difference (%)	With TD	Without TD	Difference (%)	With TD	Without TD	Difference (%)
FWHM (µm)	46.0	45.6	−0.88%	46.0	46.7	1.50%	51.3	49.4	−3.85%
Peak dose (Gray)	24.68	25.03	1.41	22.74	22.85	0.52	7.40	7.53	1.72
Valley dose (Gray)	0.83	0.85	2.07	0.89	0.90	1.24	0.41	0.42	1.40
PVDR	29.57	29.37	−0.68	25.46	25.28	−0.73	17.89	17.95	0.32
